# Mobile Phone Addiction, Interaction Anxiousness, and Eating Behavior in Nursing Students: A Moderation Analysis

**DOI:** 10.1155/jonm/3836110

**Published:** 2025-08-18

**Authors:** Hongman Li, Zhenrong Shen, Yingting Jiang, Ying Xiong, Xiaoming Sun, Yihao Zeng, Qihan Zhang, Yufei Lu, Jiagen Xiang, Zengjie Ye

**Affiliations:** ^1^School of Nursing, Guangzhou University of Chinese Medicine, Guangzhou, Guangdong, China; ^2^School of Nursing, Guangzhou Medical University, Guangzhou, Guangdong, China; ^3^Medical College of Acu-Moxi and Rehabilitation, Guangzhou University of Chinese Medicine, Guangzhou, Guangdong, China

**Keywords:** eating behavior, interaction anxiousness, latent profile analysis, mobile phone addiction, moderation analysis

## Abstract

**Background:** Mobile phone addiction could be associated with insufficient mastery of medical knowledge among nursing students, leading to a significant decline in the quality of future nursing care. Poor eating behavior among nursing students may potentially impact the quality of care they provide in future healthcare roles. This study aimed to identify different patterns of mobile phone addiction and evaluate the impact of interaction anxiousness on the association of mobile phone addiction and eating behavior.

**Methods:** Participants completed assessments including the Mobile Phone Addiction Index, Interaction Anxiousness Scale, and The Dutch Eating Behavior Questionnaire for undergraduate students. The analysis methods included latent profile analysis and examinations of moderating factors.

**Results:** Two latent mobile phone addiction types were identified: Low-mobile phone addiction (48.1%, *N* = 210) and high-mobile phone addiction (51.9%, *N* = 227). In high-mobile phone addiction subgroup, high interaction anxiousness (from −1.05 to 4.00) exacerbates the relationship between mobile phone addiction and eating behavior while high interaction anxiousness (from 3.23 to 5.03) weakens the association in low-mobile phone addiction subgroup.

**Conclusions:** Mobile phone addiction significantly affects eating behavior and nursing student exhibit heterogeneous in mobile phone addiction. Different moderation effects of interaction anxiousness are recognized in different mobile phone addiction profiles.

## 1. Introduction

Mobile phone addiction (MPA), commonly referred to mobile phone anxiety and mobile phone syndrome [1], is a behavioral addiction marked by the compulsive and excessive use of smartphones [2]. MPA can impair social functioning and contribute to the development of psychological and behavioral disorders [3]. Research indicates that 38.1% nursing students experience MPA [4]. A study revealed a negative correlation between academic achievement and the length of time spent using cellphones every day, suggesting that MPA may be associated with insufficient mastery of medical knowledge among nursing students, ultimately compromising the quality of future nursing care [5]. Additionally, the researchers identified that academic pressure, stress, social isolation, and interpersonal issues are significant contributors to MPA [6–11].

**Figure 1:**
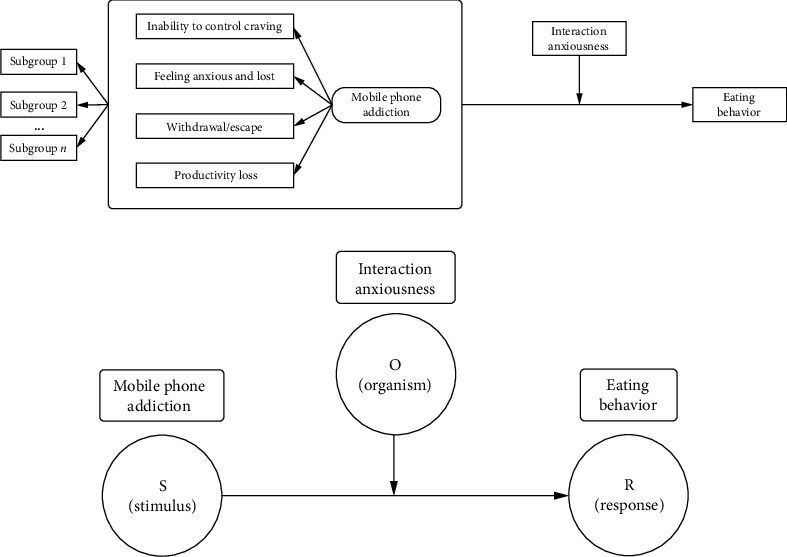
(a) The hypothetical framework of mobile phone addiction, interaction anxiousness and eating behavior among nursing students. (b) Stimulus‐organism‐response theory.

According to a recent meta-analysis, researchers found that persons who had the highest levels of Internet usage had a 47% higher likelihood of being obese compared to those with the lowest levels of Internet usage [12]. Several studies have concluded that MPA negatively impacts eating disorders and lifestyle characteristics [13, 14]. Eating disorders, such as anorexia nervosa and bulimia nervosa, are debilitating, life-threatening, and costly mental health conditions [15]. Prolonged unhealthy eating behavior can contribute to the development of eating disorders. Addressing eating behaviors is essential for preventing such conditions. Furthermore, poor eating behavior among nursing students may negatively affect their physical and emotional health, potentially impacting the quality of care they provide in future healthcare roles [16]. A previous study found a direct link between MPA and eating behaviors, including the consumption of sugar-sweetened foods. [17]. Therefore, it seems that MPA is closely related to eating behavior such as external eating and restrained eating [18]. In this study, we aimed investigate the direct relationship between MPA and eating behavior. Additionally, previous research has identified distinct patterns of MPA in nursing students [11, 19, 20]. To explore potential subgroups, we employed latent profile analysis (LPA). In nursing practice, effective communication and interaction with patients are essential. However, the interaction anxiousness of nurses may unintentionally affect their care for patients [21]. Interaction anxiousness, a frequently observed type of anxiety, encompasses strong emotional reactions and avoidance behaviors [9]. It is characterized by heightened anxiety, excessive worry, uneasiness, and occasionally fear of different interpersonal settings [22]. It is also marked by a significant fear of engaging in face-to-face interactions with others [23, 24]. As Mehrabian's Stimulus‐Organism‐Response (S-O-R) theory assumes [25], which explores how individuals respond to external stimuli (S), leading to changes in their internal organism (O), and eliciting personal response (R) accordingly [26, 27] (see Figure 1(b)), individuals with excessive smartphone addiction (S) are more likely to generate negative emotions and avoid real-world social interactions (O) [28]. Stress and negative emotions, such as interaction anxiousness, can adversely affect appetite, leading some individuals to consume more food [29]. This phenomenon is known as emotional overeating, resulting in changes in individual eating behavior (R) [30]. A previous observational study also indicated that interaction anxiousness was positively correlated with unhealthy eating behaviors [31]. Therefore, interaction anxiousness may moderate the effects of MPA on nursing students' eating behavior. Besides, we deem that under conditions of high interaction anxiousness, the impact of MPA on unhealthy eating behaviors may be intensified. For example, in the high interaction anxiousness group, this relationship is stronger. However, current research on interaction anxiousness predominantly centers on physiological mechanisms and their association with emotions [28]. In addition, the extent of MPA varies, and interaction anxiousness also affects changes in eating behaviors due to different patterns of MPA. Therefore, we propose the following hypothesis (Figure 1(a)):

1. There is a substantial correlation between MPA and eating behavior.
2. Several distinct patterns of MPA will be recognized in nursing students.
3. Interaction anxiousness moderates the relationship between MPA and eating behavior in different patterns of MPA.

## 2. Methods

### 2.1. Design and Participants

This study used moderation analysis and the LPA model in combination with a cross-sectional descriptive approach. From October 2023 to December 2023, the Be Resilient to Nursing Career (BRNC) program was conducted in Guangzhou University of Chinese Medicine. Convenience sampling was used to select 456 nursing students [24, 32–37]. The criteria for inclusion are as follows: (1) Undergraduate nursing students, and (2) voluntary to participate in this study. Exclusion criteria included clinical psychiatric diagnoses of mental disorders made by a psychiatrist. Initial data inspection found that 19 questionnaires (4.2%) had significant missing data. After excluding these cases, a total of 437 questionnaires were collected (response rate = 95.8%).

### 2.2. Sample Size

For LPA to yield reliable and accurate subgroup findings, a minimum sample size of 300 is required [38], with at least 30 participants in each subgroup [39, 40]. Wei et al. and Li et al. both indicate that with a sample size around 300, LPA can effectively identify latent classes and yield statistically significant results [40, 41]. Therefore, including 437 participants in this study provides a feasible sample size for conducting LPA.

## 3. Measures

### 3.1. Demographic Characteristics

We collected demographic characteristics including gender, grade, annual family income. In addition, data on eating behavior-related traits, such as body mass index (BMI), waist circumference, and sleep latency, were gathered based on prior studies [42–44].

### 3.2. Measurement of MPA

The MPA Index (MPAI), developed by Chinese scholar Louis Leung, was used to assess MPA [45]. The scale contains 17 items evaluating four domains: “inability to control craving”, “feeling anxious and lost”, “withdrawal/escape”, and “productivity loss” (e.g., “Your friends and family complained about your use of the mobile phone”) [46]. A 5-point Likert scale is used to score each item (1 being never and 5 being always). Higher overall ratings suggest more serious mobile phone use issues. The MPAI has demonstrated strong internal consistency [47]. In this study, we used the sum scores to measure MPA. The Cronbach's α value was 0.893.

### 3.3. Measurement of Interaction Anxiousness

The Interaction Anxiousness Scale (IAS) was used to assess tendencies towards subjective social anxiety experiences independent of behaviors [48]. The IAS comprises 15 self-report items, each rated on a 5-point scale ranging from 1 (not at all like me) to 5 (very like me). These items were chosen based on two criteria: (1) They assess subjective feelings of anxiety (such as nervousness and neuroticism) or their opposites (like relaxation and calmness), focusing purely on internal experiences rather than observable behaviors. (2) They measure responses in unforeseen social scenarios where individuals' reactions are influenced by the presence or responses of others, distinct from situations involving more predictable social dynamics, such as public speaking [49]. After going through four stages of development, the scale's original selection of 87 elements was reduced to its present set of 15. The scale's total scores range from 15 (the lowest) to 75 (the highest) [50]. The Cronbach's α value was 0.820.

### 3.4. Measurement of Eating Behavior

The Dutch Eating Behavior Questionnaire (DEBQ) was used to assess eating behavior [51]. The main emphasis lies in the examination of eating patterns influenced by adverse emotions. Respondents provide ratings for 33 items, ranging from “never” to “very often” [52]. The Chinese version of the DEBQ was translated by Wang, a Taiwanese scholar [53]. The questionnaire includes subscales for emotional eating, external eating, and restrained eating, comprising 13, 10, and 10 items, respectively, all demonstrating strong internal consistency [54]. In this study, total scores were used to assess eating behavior, with a Cronbach's α of 0.939.

### 3.5. Data Analyses

Initially, the calculation of descriptive statistics was performed, encompassing frequencies, percentages, means, and standard deviations. Subsequently, variations among subgroups were analyzed using independent sample *t*-tests and analysis of variance (ANOVA) [55]. In the second step, the relationships among MPA, interaction anxiousness, and eating behavior were investigated through Pearson correlation analysis. Thirdly, LPA was employed to identify different subgroups with different levels of MPA [56–58]. Model fit and the difference between the expected and observed values were assessed using Akaike Information Criterion (AIC), Bayesian Information Criterion (BIC), and sample size-adjusted BIC (aBIC) [59]. Fourthly, comparisons between eating behavior and various LPA-based MPA profiles were conducted using Independent samples *t*-test [60]. Finally, interaction anxiousness was assessed as a moderating factor in the relationship between distinct patterns of MPA and eating behavior. The Johnson–Neyman moderation analysis was applied to describe the range, direction, and magnitude of the moderation effects more precisely [61].

The data processing tools used were SPSS 26.0, Mplus 8.3, and JASP 0.18.3. The significance level was set at 0.05.

### 3.6. Ethical Considerations

The research was granted approval by the Ethics Board of the First Affiliated Hospital of Guangzhou University of Chinese Medicine (ZYYEC-ERK[2020]132). Each participant provided informed consent after receiving a verbal description of the procedure and its objective, in compliance with the principles stated in the Declaration of Helsinki. The participants were given assurances regarding the confidentiality of their data and the preservation of their anonymity.

## 4. Results

### 4.1. Demographic Characteristics

The overall sample comprised 437 nursing students, of which 359 (82.2%) were female. Their average age was 19.23 (SD = 0.87). Only 22.0% of the nursing students were only-child and the majority of students (51.3%) had family income ranging from 80,000 to 150,000 RMB per year. Additional demographic information is provided in Table 1.

### 4.2. Correlation Analysis of MPA, Interaction Anxiousness, and Eating Behavior

**Figure 2:**
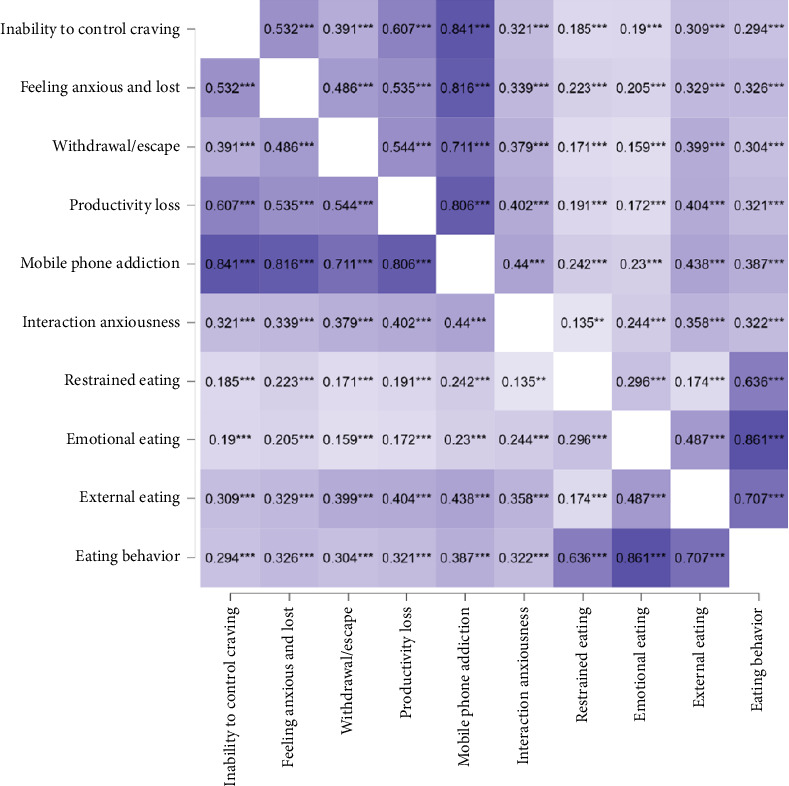
Pearson correlation heatmap among mobile phone addiction, interaction anxiousness and eating behavior. Note: ^∗^*p* < 0.05, ^∗∗^*p* < 0.01, ^∗∗∗^*p* < 0.001.

The average and standard deviations of each variable were: inability to control craving (18.25 ± 4.95), feeling anxious and lost (11.03 ± 4.13), withdrawal/escape (9.55 ± 3.01), MPA (48.85 ± 11.85), interaction anxiousness (44.00 ± 8.98), restrained eating (24.65 ± 8.98), emotional eating (32.30 ± 12.08), external eating (33.23 ± 7.74), and eating behavior (90.17 ± 21.39). There was a strong correlation between MPA and interaction anxiousness (*r* = 0.44), indicating a substantial association. Additionally, MPA was positively correlated with eating behavior (*r* = 0.39). Furthermore, there was also a positive relationship between interaction anxiousness and eating behavior (*r* = 0.32). However, the correlation between interaction anxiousness and restrained eating was weak (*r* = 0.14). Additional information is provided in the Pearson correlation heatmap (Figure 2).

### 4.3. LPA of MPA Traits

**Figure 3:**
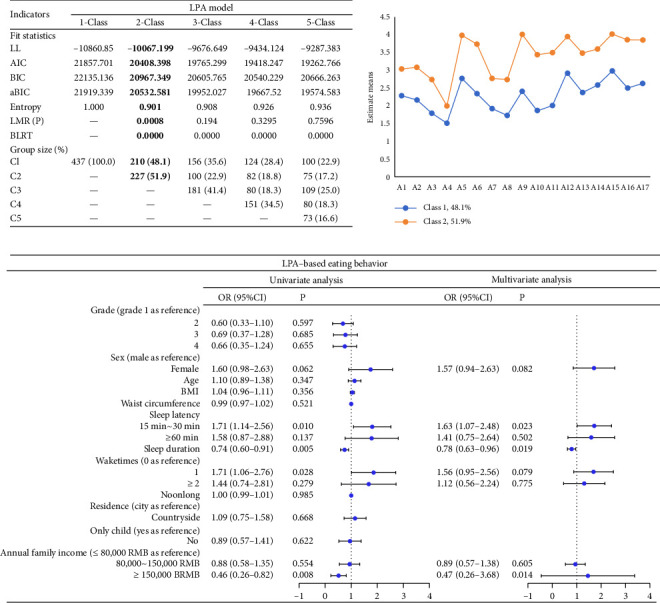
(a) Fitting index and group size of latent profile analysis models. Note: bold figures highlight the selected class solution. (b) Parameters for the final two-class patterns. C1 = low-mobile phone addiction, C2 = high-mobile phone addiction. Note: The *x*-axis represents the 17 items of MPA, the *y*-axis shows the estimated mean for each item. (c) Univariate and multivariate logistic regression results for predicting external features on the 2-class pattern.

We examined from 1 to 5 latent groups using AIC, BIC, aBIC, and other criteria. As model complexity increased, the AIC and BIC values tended to decrease but did not reach a significant minimum. Incorporating the three subgroups profile rendered the Lo–Mendell–Rubin likelihood ratio test nonsignificant. Hence, the optimal model was determined to be the configuration of two subgroups (AIC = 20,408, BIC = 20,967, *p* = 0.0008), selected based on theoretical considerations and simplicity. Additional details are provided in Figures 3(a), 3(b). Two MPA subgroups were identified: Low-MPA (Class 1, 48.1%, *N* = 210), and high-MPA (Class 2, 51.9%, *N* = 227). Therefore, hypothesis 1 was confirmed. Both univariate and multivariate logistic regression analyses indicated that sleep latency (OR = 1.63, 95% CI: 1.07–2.48, *p* = 0.023), measured by participants' self-reported time to fall asleep on the questionnaire, was a significant factor in distinguishing between Profile 1 and Profile 2 (as shown in Figure 3(c)).

### 4.4. LPA-Based MPA Differences in Eating Behavior Scores

Independent samples *t*-test was used to explore LPA-based MPA differences in eating behavior scores. The eating behavior average scores of low-MPA was 83.84 (SD = 21.11). The eating behavior average scores of high-MPA was 96.04 (SD = 19.97). The results depicted in Table 2 indicate that there were significant differences (*t* = −6.206, *p* < 0.001) in the eating behavior ratings between those with low-MPA and those with high-MPA. The independent samples *t*-test, as shown in Table 2, verified these conclusions.

### 4.5. Moderation Analysis of Interaction Anxiousness Between Distinct Patterns of MPA and Eating Behavior

**Figure 4:**
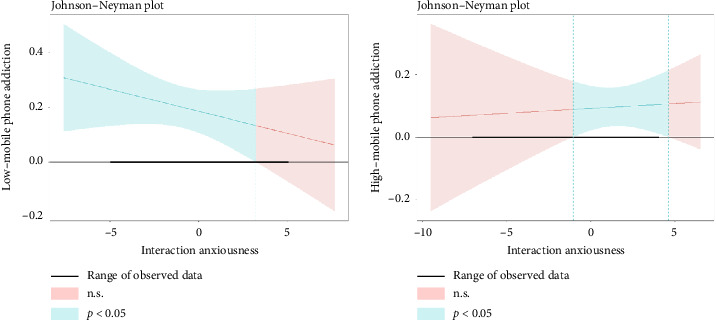
(a) Johnson-Neyman plot of low-mobile phone addiction on eating behavior. (b) Johnson-Neyman plot of high-mobile phone addiction on eating behavior.

Before starting the analysis, gender was included as a control variable due to the imbalance in the gender ratio of the sample. In low-MPA subgroup, the values obtained were as follows: under the moderation of high interaction anxiousness (from 3.23 to 5.03), the relationship between MPA and eating behavior was weaken (as shown in Figure 4(a)). In high-MPA subgroup, the values obtained were as follows: under the moderation of high interaction anxiousness (from −1.05 to 4.00), MPA-eating behavior showed positive corrections (as shown in Figure 4(b)).

## 5. Discussion

In this study, MPA significantly affected eating behavior and nursing students exhibit heterogeneous levels of MPA. Different moderation effects of interaction anxiousness are recognized in different MPA profiles.

First, consistent with prior research, the current study found a positive correlation between MPA and eating behavior, supporting Hypothesis 1 [18, 62, 63]. In China, nursing students face a heavy academic workload and using mobile phones has become their primary leisure activity [64]. Consequently, other recreational pursuits are often replaced by unhealthy eating behavior, for example, binge eating [65]. However, such behavior can impact the health of nursing students and consequently affect the quality of care they provide to patients in the future [66]. Therefore, college educators should promote a healthy attitude toward eating.

Second, MPA among nursing students can be categorized into two subgroups by LPA, named as low-MPA (48.1%) and high-MPA (51.9%), with nearly equal numbers in both groups. High-MPA is more likely to be associated with unhealthy eating behavior and warrants attention. Therefore, this finding is consistent with Hypothesis 2. In addition, we performed binary logistic regression analysis on both subgroups. Nursing students from families earning more than 15,000 RMB per year were less likely to be addicted to mobile phone compared to those earning less than 8000 RMB per year. Previous research has indicated that parents from low-income families may lack sufficient time to supervise their children's mobile phone usage, potentially leading to higher risk of MPA among nursing students from lower-income households [67]. Furthermore, because of reduced incomes for families, several nursing students choose mobile phones as a financially efficient means of entertainment [68]. For example, while affluent individuals can embark on travel without hesitation, students from low-income households may rely on watching short videos on their mobile phones to experience scenic beauty. This exacerbates their susceptibility to MPA. Moreover, this study revealed that nursing students who experienced prolonged sleep latency were more susceptible to MPA, aligning with prior research findings [8, 69, 70]. Some reports suggested that excessive mobile phone usage before bedtime can adversely affect sleep quality [71]. Additionally, Loughran et al. documented the adverse impacts of electromagnetic fields radiated by phones on sleep electroencephalography [72, 73].

Third, this study found that for nursing students with high-mobile addiction, high interaction anxiousness may exacerbate unhealthy eating behaviors. However, for nursing students with low-mobile addiction, high interaction anxiousness may weaken the relationship. Although the different patterns of MPA were statistically different, we found that interaction anxiousness moderates the relationship between different patterns of MPA and eating behavior within a certain range, supporting Hypothesis 3. The findings revealed that MPA had an impact on the eating behavior of nursing students, and it was also observed that interaction anxiousness played a role in influencing eating behavior, and the moderation effect of interaction anxiousness was significant within a certain range. On the one hand, for nursing students with high-mobile addiction, high interaction anxiousness may strengthen unhealthy eating behaviors, which is consistent with the previous studies [43, 74]. Nursing students with high-mobile addiction have less time for socializing [75]. Consequently, they generally experience more severe interaction anxiousness issues [76]. Nursing students with higher levels of interaction anxiousness may lead them to redirect their social energy towards unhealthy eating habits [77]. Therefore, colleges should organize regular social activities and provide psychological counseling to help nursing students with high-MPA alleviate interaction anxiousness. On the other hand, for nursing students with low-mobile addiction, high interaction anxiousness may weaken the relationship. Research shows that in high-anxiety states, individuals with higher levels of addiction are more likely to adopt unhealthy coping strategies, such as binge eating [78]. Therefore, nursing students with low levels of mobile addiction may be more likely to engage in positive coping strategies, such as running and getting an early night's sleep, to manage their anxiety related to interactions.

## 6. Implications for Nursing Practice

In nursing education and student support services, it is essential to identify nursing students with high-MPA first, as these students are more likely to exhibit unhealthy eating behaviors. This study found that students from low-income families and those with prolonged sleep latency are more susceptible to MPA. Therefore, nursing educators should pay more attention to students from low-income backgrounds and those with prolonged sleep latency to better identify nursing students with high MPA. Furthermore, for nursing students with high-MPA, higher interaction anxiousness may exacerbate unhealthy eating behaviors. Therefore, it is crucial to implement measures to alleviate their interaction anxiousness, such as offering mindfulness meditation courses to help reduce their anxiety [77].

## 7. Limitations

This study inevitably has certain limitations. On the one hand, the study sample is limited to undergraduate nursing students from a university in China, which may not be representative and carries the risk of selection bias. Therefore, future studies should use samples with diverse backgrounds to further validate the findings. On the other hand, as a cross-sectional study, it cannot establish causality. Our research team will focus on conducting longitudinal research in the future.

## 8. Conclusion

MPA significantly affects eating behavior and nursing students exhibit heterogeneous in MPA. Different moderation effects of interaction anxiousness are recognized in different MPA profiles. Nursing instructors and educators ought to promptly identify MPA in students and provide support to alleviate interaction anxiousness and promote healthier eating behavior.

## Figures and Tables

**Table 1:** Demographic analysis among nursing students.

Variables	Outcome variable: eating behavior		
	M ± SD	*n* (%)	*p* value
Age	19.23 ± 0.87		
BMI	20.29 ± 2.61		
Waist circumference	67.78 ± 8.76		
Sleep duration	9.92 ± 0.95		
Grade			0.229
1	95.31 ± 21.54	62 (14.2%)	
2	89.42 ± 21.87	144 (33.0%)	
3	88.74 ± 18.24	123 (28.1%)	
4	89.86 ± 21.39	108 (24.7%)	
Sex			0.001
Male	82.65 ± 21.35	78 (17.8%)	
Female	91.81 ± 21.08	359 (82.2%)	
Sleep latency			0.574
≤ 15 min	89.23 ± 19.95	200 (45.8%)	
15–30 min	91.45 ± 1.63	182 (41.6%)	
≥ 60 min	89.40 ± 24.43	55 (12.6%)	
Waketimes			0.068
0	89.60 ± 21.25	306 (70.0%)	
1	91.27 ± 20.04	91 (20.8%)	
≥ 2	92.08 ± 25.49	40 (9.2%)	
Residence			0.221
City	88.92 ± 22.59	218 (49.9%)	
Countryside	91.42 ± 20.11	219 (50.1%)	
Only child			0.727
Yes	89.50 ± 22.19	96 (22.0%)	
No	90.36 ± 21.19	341 (78.0%)	
Annual family income			0.679
≤ 80,000 RMB	91.71 ± 20.38	141 (32.3%)	
80,000–150,000 RMB	89.82 ± 22.38	224 (51.3%)	
≥ 150,000 BRMB	88.26 ± 20.27	72 (16.5%)	

**Table 2:** Independent sample *t*-test of eating behavior scores across LPA-based mobile phone addiction types.

LPA-based differences in eating behavior scores				
Subgroups	*N*	M ± SD	*t*	*p*
Low-mobile phone addiction	210	83.84 ± 21.11	−6.206	< 0.001
High-mobile phone addiction	227	96.04 ± 19.97		

## Data Availability

The data that support the findings of this study are available from the corresponding authors upon reasonable request.

## References

[1] Ma H., He J. Q., Zou J. M., Zhong Y. (2021). Mobile Phone Addiction and its Association With Burnout in Chinese Novice Nurses: A Cross-Sectional Survey. *Nursing Open*.

[2] Xiao W., Zhou H., Li X., Lin X. (2021). Why Are Individuals With Alexithymia Symptoms More Likely to Have Mobile Phone Addiction? The Multiple Mediating Roles of Social Interaction Anxiousness and Boredom Proneness. *Psychology Research and Behavior Management*.

[3] Song A., Song G., Wang H. (2023). Prevalence of Mobile Phone Addiction Among Medical Students: A Systematic Review. *American Journal of Tourism Research*.

[4] Zhou B., Mui L. G., Li J., Yang Y., Hu J. (2024). A Model for Risk Factors Harms and of Smartphone Addiction Among Nursing Students: A Scoping Review. *Nurse Education in Practice*.

[5] Kalal N., Vel N. S., Angmo S. (2023). Smartphone Addiction and its Impact on Quality of Sleep and Academic Performance Among Nursing Students. Institutional Based Cross-Sectional Study in Western Rajasthan (India). *Investigación y Educación en Enfermería*.

[6] Liu M., Lu C. (2022). Mobile Phone Addiction and Depressive Symptoms Among Chinese University Students: The Mediating Role of Sleep Disturbances and the Moderating Role of Gender. *Frontiers in Public Health*.

[7] Li Y., Li G., Liu L., Wu H. (2020). Correlations Between Mobile Phone Addiction and Anxiety, Depression, Impulsivity, and Poor Sleep Quality Among College Students: A Systematic Review and Meta-Analysis. *Journal of Behavioral Addictions*.

[8] Nikolic A., Bukurov B., Kocic I. (2023). Smartphone Addiction, Sleep Quality, Depression, Anxiety, and Stress Among Medical Students. *Frontiers in Public Health*.

[9] Tateno M., Teo A. R., Ukai W. (2019). Internet Addiction, Smartphone Addiction, and Hikikomori Trait in Japanese Young Adult: Social Isolation and Social Network. *Frontiers in Psychiatry*.

[10] Zhang Y., Li Y., Xia M., Han M., Yan L., Lian S. (2023). The Relationship Between Loneliness and Mobile Phone Addiction Among Chinese College Students: The Mediating Role of Anthropomorphism and Moderating Role of Family Support. *PLoS One*.

[11] Cheng C., Ebrahimi O. V., Luk J. W. (2022). Heterogeneity of Prevalence of Social Media Addiction Across Multiple Classification Schemes: Latent Profile Analysis. *Journal of Medical Internet Research*.

[12] Aghasi M., Matinfar A., Golzarand M., Salari-Moghaddam A., Ebrahimpour-Koujan S. (2020). Internet Use in Relation to Overweight and Obesity: A Systematic Review and Meta-Analysis of Cross-Sectional Studies. *Advances in Nutrition*.

[13] Morse K. L., Driskell J. A. (2009). Observed Sex Differences in Fast-Food Consumption and Nutrition Self-Assessments and Beliefs of College Students. *Nutrition Research*.

[14] Kim Y., Park J. Y., Kim S. B., Jung I. K., Lim Y. S., Kim J. H. (2010). The Effects of Internet Addiction on the Lifestyle and Dietary Behavior of Korean Adolescents. *Nutrition Research and Practice*.

[15] Treasure J., Duarte T. A., Schmidt U. (2020). Eating Disorders. *The Lancet*.

[16] Vitale E., Mea R. (2024). Comorbidity, Eating Behaviors and Smartphone Addiction in Italian Nurses’ Characteristics. *Endocrine, Metabolic & Immune Disorders—Drug Targets*.

[17] Liu S., Zhou W., Wang J., Chen B., He G., Jia Y. (2021). Association Between Mobile Phone Addiction Index and Sugar-Sweetened Food Intake in Medical College Students Stratified by Sex from Shanghai, China. *Nutrients*.

[18] Panea-Pizarro I., López-Espuela F., Martos-Sánchez A., Domínguez-Martín A. T., Beato-Fernández L., Moran-García J. M. (2020). Internet Addiction and Facebook Addiction in Spanish Women With Eating Disorders. *Archives of Psychiatric Nursing*.

[19] Drossel G., Brucar L. R., Rawls E., Hendrickson T. J., Zilverstand A. (2023). Subtypes in Addiction and Their Neurobehavioral Profiles Across Three Functional Domains. *Translational Psychiatry*.

[20] Kim B. N., Kang H. S., Park J. (2023). A Latent Profile Approach for Classifying Internet Gamers Based on Motives for Online Gaming. *Journal of Behavioral Addictions*.

[21] Hofmann S. G., Asnaani A., Vonk I. J., Sawyer A. T., Fang A. (2012). The Efficacy of Cognitive Behavioral Therapy: A Review of Meta-Analyses. *Cognitive Therapy and Research*.

[22] Zhou H., Xiao W., Li X., Jiang H. (2022). The Influence of Alexithymia on Problematic Mobile Phone Use Among Chinese Adolescent Students: Multiple Mediating Roles of Social Interaction Anxiousness and Core Self-Evaluations. *Journal of Affective Disorders*.

[23] Morrison A. S., Heimberg R. G. (2013). Social Anxiety and Social Anxiety Disorder. *Annual Review of Clinical Psychology*.

[24] Mei X. X., Wu X. N., Wang H. Y., Wu J. Y., Wang X. Q., Ye Z. J. (2022). Heterogeneity in Psychological Resilience and Mental Health Among Newly Graduated Nursing Students: A Latent Profile and Generalized Additive Model Analysis. *Psychology Research and Behavior Management*.

[25] Mehrabian A., Russell J. A. (1974). *An Approach to Environmental Psychology*.

[26] Nian S., Li D., Zhang J., Lu S., Zhang X. (2023). Stimulus-Organism-Response Framework: Is the Perceived Outstanding Universal Value Attractiveness of Tourists Beneficial to World Heritage Site Conservation?. *International Journal of Environmental Research and Public Health*.

[27] Li M., Wang Q., Cao Y. (2022). Understanding Consumer Online Impulse Buying in Live Streaming E-Commerce: A Stimulus-Organism-Response Framework. *International Journal of Environmental Research and Public Health*.

[28] Reid D. J., Reid F. J. M. (2007). Text or Talk? Social Anxiety, Loneliness, and Divergent Preferences for Cell Phone Use. *CyberPsychology and Behavior*.

[29] Levinson C. A., Brosof L. C., Vanzhula I. (2018). Social Anxiety and Eating Disorder Comorbidity and Underlying Vulnerabilities: Using Network Analysis to Conceptualize Comorbidity. *International Journal of Eating Disorders*.

[30] Dakanalis A., Mentzelou M., Papadopoulou S. K. (2023). The Association of Emotional Eating With Overweight/Obesity, Depression, Anxiety/Stress, and Dietary Patterns: A Review of the Current Clinical Evidence. *Nutrients*.

[31] Dougherty E. N., Johnson N. K., Badillo K., Haedt-Matt A. A. (2023). Sleep Reactivity is Associated With Social Anxiety and Disordered-Eating Behaviors in College Students. *Journal of American College Health*.

[32] Mei X. X., Wang H. Y., Wu X. N., Wu J. Y., Lu Y. Z., Ye Z. J. (2022). Self-Efficacy and Professional Identity Among Freshmen Nursing Students: A Latent Profile and Moderated Mediation Analysis. *Frontiers in Psychology*.

[33] Wu X., Lu Y., Zhang Q. (2022). Stress/Resource Complex, Sense of Coherence and Professional Identity Among Nursing Students: A Latent Profile and Mediation Analysis. *Psychology Research and Behavior Management*.

[34] Mei X., Wang H., Wang X., Wu X., Wu J., Ye Z. (2022). Associations Among Neuroticism, Self-Efficacy, Resilience and Psychological Distress in Freshman Nursing Students: A Cross-Sectional Study in China. *BMJ Open*.

[35] Li Z.-S., Hasson F. (2020). Resilience, Stress, and Psychological Well-Being in Nursing Students: A Systematic Review. *Nurse Education Today*.

[36] Wu X., Lu Y., Xie X. (2022). Association Between Circadian Rhythm and Sleep Quality Among Nursing Interns: A Latent Profile and Moderation Analysis. *Frontiers in Neuroscience*.

[37] Wu X., Lu Y., Zeng Y. (2024). Personality Portraits, Resilience, and Professional Identity Among Nursing Students: A Cross-Sectional Study. *BMC Nursing*.

[38] Li S., Wang X., Wang M. (2023). Association Between Stigma and Sleep Quality in Patients With Breast Cancer: A Latent Profile and Mediation Analysis. *European Journal of Oncology Nursing*.

[39] Dalmaijer E. S., Nord C. L., Astle D. E. (2022). Statistical Power for Cluster Analysis. *BMC Bioinformatics*.

[40] Li S., Xiang Y., Li H. (2024). Body Image, Self-Efficacy, and Sleep Quality Among Patients With Breast Cancer: A Latent Profile and Mediation Analysis. *European Journal of Oncology Nursing*.

[41] Wei M., Mallinckrodt B., Arterberry B. J., Liu S., Wang K. T. (2021). Latent Profile Analysis of Interpersonal Problems: Attachment, Basic Psychological Need Frustration, and Psychological Outcomes. *Journal of Counseling Psychology*.

[42] Ayran G., Süleyman Z., Avcı Ü., Arık U. (2021). The Effect of Internet Addiction on Eating Attitude and Body Image in University Students. *Journal of Child and Adolescent Psychiatric Nursing: Official Publication of the Association of Child and Adolescent Psychiatric Nurses, Inc*.

[43] Banna M. H. A., Akter S., Kabir H. (2023). Internet Addiction, Depressive Symptoms, and Anxiety Symptoms are Associated With the Risk of Eating Disorders Among University Students in Bangladesh. *Scientific Reports*.

[44] Mohammad Johari M. H., Tan S. T. (2024). Internet Addiction and its Relationship With Food Choice Motives and the Risk of Eating Disorders Among Young Adults in Malaysia. *Scientific Reports*.

[45] Leung L. (2008). Linking Psychological Attributes to Addiction and Improper Use of the Mobile Phone Among Adolescents in Hong Kong. *Journal of Children and Media*.

[46] Yang L. L., Guo C., Li G. Y., Gan K. P., Luo J. H. (2023). Mobile Phone Addiction and Mental Health: The Roles of Sleep Quality and Perceived Social Support. *Frontiers in Psychology*.

[47] Wang R., Yang R., Ran H. (2022). Mobile Phone Addiction and Non-Suicidal Self-Injury Among Adolescents in China. *PeerJ*.

[48] Leary M. R. (1983). Social Anxiousness: The Construct and its Measurement. *Journal of Personality Assessment*.

[49] He X. (2022). Relationship Between Self-Esteem, Interpersonal Trust, and Social Anxiety of College Students. *Occupational Therapy International*.

[50] Chunzi P., Yaoxian G., Xiongzhao Z. (2004). Reliability and Validity of the Social Anxiety Scale: Applicability Among Chinese University Students. *Chinese Journal of Mental Health*.

[51] Tv S., Frijters J., Bergers G. P. A., Defares P. B. (1986). The Dutch Eating Behavior Questionnaire (DEBQ) for Assessment of Restrained, Emotional, and External Eating Behavior. *International Journal of Geographical Information Science*.

[52] Benbaibeche H., Saidi H., Bounihi A., Koceir E. A. (2023). Emotional and External Eating Styles Associated With Obesity. *Journal of Eating Disorders*.

[53] Wang Y. F., Chuang H. L., Chang C. W., Zauszniewski J. A. (2017). Translation and Psychometric Properties of a Chinese Version of the Dutch Eating Behavior Questionnaire for Children in Taiwanese Preadolescents. *Western Journal of Nursing Research*.

[54] Wang Y. F., Ha S., Zauszniewski J. A., Ross R. (2018). Psychometric Properties of the Chinese Version of the Dutch Eating Behavior Questionnaire in a Sample of Taiwanese Parents. *Obesity Research & Clinical Practice*.

[55] Cohen J. (1977). *Statistical Power Analysis for the Behavioral Sciences*.

[56] Zhao Z., Mei Y., Wang X. (2023). Meaning in Life Among Nursing Students: A Latent Profile Analysis. *BMC Nursing*.

[57] Teng M., Wang J., Jin M. (2023). Psychological Capital Among Clinical Nurses: A Latent Profile Analysis. *International Nursing Review*.

[58] Journault A. A., Plante I., Charbonneau S. (2022). Using Latent Profile Analysis to Uncover the Combined Role of Anxiety Sensitivity and Test Anxiety in Students’ State Anxiety. *Frontiers in Psychology*.

[59] Berlin K. S., Williams N. A., Parra G. R. (2014). An Introduction to Latent Variable Mixture Modeling (Part 1): Overview and Cross-Sectional Latent Class and Latent Profile Analyses. *Journal of Pediatric Psychology*.

[60] Kelter R. (2020). Bayesian Alternatives to Null Hypothesis Significance Testing in Biomedical Research: A Non-Technical Introduction to Bayesian Inference With JASP. *BMC Medical Research Methodology*.

[61] Bauer D. J., Curran P. J. (2005). Probing Interactions in Fixed and Multilevel Regression: Inferential and Graphical Techniques. *Multivariate Behavioral Research*.

[62] Park E. J., Hwang S. S., Lee M. S., Bhang S. Y. (2022). Food Addiction and Emotional Eating Behaviors Co-occurring With Problematic Smartphone Use in Adolescents?. *International Journal of Environmental Research and Public Health*.

[63] Örnek B. Y., Gündoğmuş İ. (2022). The Effects of Smartphone and Internet Gaming Addiction on Eating Attitudes Among University Students. *Psychiatry Investigation*.

[64] Liu J., Yu X., Kong L., Zhou X. (2023). Prevalence and Factors Associated With Smartphone Addiction Among Nursing Postgraduates During the COVID-19 Pandemic: A Multilevel Study From China’s Mainland. *BMC Psychiatry*.

[65] Wang J., Hao Q. H., Peng W., Tu Y., Zhang L., Zhu T. M. (2023). Relationship Between Smartphone Addiction and Eating Disorders and Lifestyle Among Chinese College Students. *Frontiers in Public Health*.

[66] Osorio-Molina C., Martos-Cabrera M. B., Membrive-Jiménez M. J. (2021). Smartphone Addiction, Risk Factors and Its Adverse Effects in Nursing Students: A Systematic Review and Meta-Analysis. *Nurse Education Today*.

[67] Lee J., Lim H., Allen J., Choi G., Jung J. (2021). Smartphone Addiction and Depression Among Low-Income Boys Since COVID-19: The Moderating Effect of Being an Only Child. *Health Care*.

[68] Aktürk Ü., Budak F., Gültekin A., Özdemir A. (2018). Comparison of Smartphone Addiction and Loneliness in High School and University Students. *Perspectives in Psychiatric Care*.

[69] Rathakrishnan B., Bikar Singh S. S., Kamaluddin M. R. (2021). Smartphone Addiction and Sleep Quality on Academic Performance of University Students: An Exploratory Research. *International Journal of Environmental Research and Public Health*.

[70] Demirci K., Akgönül M., Akpinar A. (2015). Relationship of Smartphone Use Severity With Sleep Quality, Depression, and Anxiety in University Students. *Journal of Behavioural Addictions*.

[71] Kwon M., Kim D. J., Cho H., Yang S. (2013). The Smartphone Addiction Scale: Development and Validation of a Short Version for Adolescents. *PLoS One*.

[72] Loughran S. P., Wood A. W., Barton J. M., Croft R. J., Thompson B., Stough C. (2005). The Effect of Electromagnetic Fields Emitted by Mobile Phones on Human Sleep. *NeuroReport*.

[73] Cain N., Gradisar M. (2010). Electronic Media Use and Sleep in School-Aged Children and Adolescents: A Review. *Sleep Medicine*.

[74] Zhao M., Huang Y., Wang J., Feng J., Zhou B. (2023). Internet Addiction and Depression Among Chinese Adolescents: Anxiety as a Mediator and Social Support as a Moderator. *Psychology Health & Medicine*.

[75] Xu C., Yang X. (2019). The Research Progress on Social Anxiety in Nursing Students. *Chinese General Practice Nursing*.

[76] Ghawadra S. F., Lim Abdullah K., Choo W. Y., Danaee M., Phang C. K. (2020). The Effect of Mindfulness-Based Training on Stress, Anxiety, Depression and Job Satisfaction Among Ward Nurses: A Randomized Control Trial. *Journal of Nursing Management*.

[77] Stinson C., Curl E. D., Hale G. (2020). Mindfulness Meditation and Anxiety in Nursing Students. *Nursing Education Perspectives*.

[78] Fang L., Xu X., Lin X. (2019). Association of Mobile Phone Overuse With Sleep Disorder and Unhealthyeating Behaviors in College Students of a Medical University in Guangzhou. *Journal of Southern Medical University*.

